# Ethanolic extract of *Nymphaea lotus* L. (Nymphaeaceae) leaves exhibits in vitro antioxidant, in vivo anti-inflammatory and cytotoxic activities on Jurkat and MCF-7 cancer cell lines

**DOI:** 10.1186/s12906-020-03195-w

**Published:** 2021-01-07

**Authors:** Benoit Banga N’guessan, Adwoa Dede Asiamah, Nana Kwame Arthur, Samuel Frimpong-Manso, Patrick Amoateng, Seth Kwabena Amponsah, Kennedy Edem Kukuia, Joseph Adusei Sarkodie, Kwabena Frimpong-Manso Opuni, Isaac Julius Asiedu-Gyekye, Regina Appiah-Opong

**Affiliations:** 1 Department of Pharmacology and Toxicology, School of Pharmacy, College of Health Sciences, University of Ghana, P.O. Box LG 43, Legon, Accra, Ghana; 2 Department of Pharmaceutical Chemistry, School of Pharmacy, College of Health Sciences, University of Ghana, P.O. Box LG 43, Legon, Accra, Ghana; 3 Department of Pharmacognosy and Herbal Medicine, School of Pharmacy, College of Health Sciences, University of Ghana, P.O. Box LG 43, Legon, Accra, Ghana; 4 Department of Clinical Pathology, Noguchi Memorial Institute for Medical Research (NMIMR), College of Health Sciences, University of Ghana, Accra, Ghana

**Keywords:** *Nymphaea lotus*, Cytotoxicity, Micro/macro-elements, MCF7, Jurkat

## Abstract

**Background:**

*Nymphaea lotus* L. *(N. lotus)* is an aquatic plant with anecdotal reports suggesting its use in the traditional management of cancer. However, there is a paucity of data on the antioxidant, anti-inflammatory and cytotoxic properties of *N. lotus* in relation to its phytochemical and elemental contents. This study aimed at determining the antioxidant, anti-inflammatory and cytotoxic properties of the hydro-ethanolic extract of *N. lotus* leaves (NLE), and its phenolic, flavonoid and elemental constituents.

**Methods:**

The antioxidant property of NLE was determined using total phenolic and flavonoid, DPPH radical scavenging, lipid peroxidation and reducing power assays. The anti-inflammatory activity of NLE (100–250-500 mg/kg), diclofenac and hydrocortisone (positive controls) were determined by paw oedema and skin prick tests in *Sprague Dawley* rats. Also, the erythrocyte sedimentation rate (ESR) was determined by Westergren method. The macro/micro-elements content was determined by the XRF method. The cytotoxic property of NLE was determined by the MTT assay, on two cancer cell lines (MCF-7 and Jurkat) and compared to a normal cell line (Chang liver). Inhibitory concentrations were determined as IC_50_ values (±SEM).

**Results:**

The extract had appreciable levels of phenolic and flavonoids compounds and was two-fold more potent in scavenging DPPH radicals than Butylated hydroxytoluene (BHT). However, NLE was three- and six-fold less potent than ascorbic acid and BHT, respectively, in reducing Fe^3+^ to Fe^2+^. The extract was six-fold more potent than gallic acid in inhibiting lipid peroxidation. The extract caused a dose-dependent decrease in rat paw oedema sizes, comparable to diclofenac, and a significant decrease in wheel diameters and ESR. The elemental analysis revealed relevant concentrations of Mg^2+^, P^2+^, S^2+^, K^2+^, Mn^+^, Fe^+^, Cu^+^, Zn^+^ and Cd^+^. The extract exhibited cytotoxic activity on both MCF-7 (IC_50_ = 155.00 μg/ml) and Jurkat (IC_50_ = 87.29 μg/ml), with higher selectivity for Jurkat cell line. Interestingly, the extract showed low cytotoxicity to the normal Chang liver cell line (IC_50_ = 204.20 μg/ml).

**Conclusion:**

*N. lotus* leaves extract exhibited high antioxidant, anti-inflammatory and cancer-cell-specific cytotoxic properties. These aforementioned activities could be attributed to its phenolic, flavonoid and elemental constituents.

**Graphical abstract:**

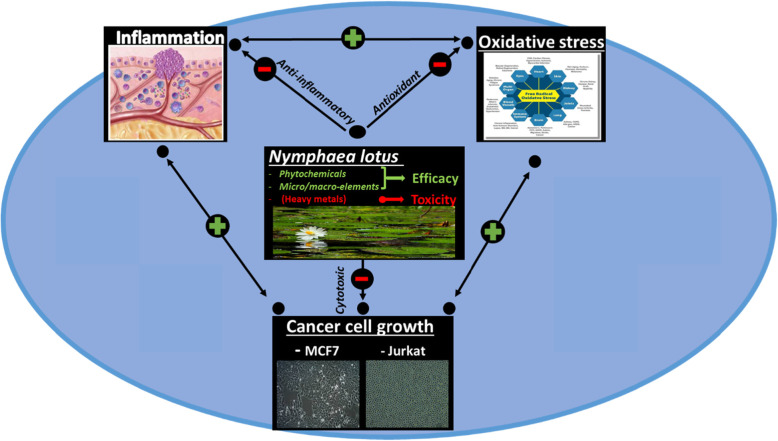

## Background

Non-communicable diseases (NCDs) such as cardiomyopathies, chronic respiratory diseases, diabetes, and cancer are among the commonest causes of mortality and morbidity worldwide. In sub-Saharan-Africa, NCDs represent a significant cause of death and disability. NCDs are predicted to surpass infectious diseases as the most important cause of morbidity and mortality by 2030 [1–3]. Oxidative stress (OS) and inflammation are primary molecular mechanisms that play critical roles in the onset and development of NCDs [4, 5]. Understanding the role of OS and inflammation in NCDs has paved way for the discovery of novel compounds with high antioxidant and anti-inflammatory potential, that can be used in the management of NCDs and cancer in particular.

Cancer is reported to be the second leading cause of death worldwide, with an estimated 9.6 million deaths in 2018 [6, 7]. This estimation has been projected to double by the next coming decade despite therapeutic advances made in recent years and the improvement in the management of cancer patients [7, 8]. It is noteworthy, that, nearly 70% of these cancer-related deaths happen in low- and middle-income countries (https://www.who.int/news-room/fact-sheets/detail/cancer). According to the New Global Cancer Data [6], Africa has the highest proportion of cancer deaths compared with its incidence, and this is likely due to the high occurrence of cancer types related to poor prognosis along with restricted access to timely diagnosis and treatment [9]. Examples of common and/or neglected cancers are breast cancer and leukaemia.

Breast cancer is a common invasive cancer found in females. Data suggests that prevalence of breast cancer is on the increase among Ghanaian women, unlike in North American women [10, 11]. Currently, a surrogate classification of five subtypes of breast cancer (based on histological and molecular characteristics) is being used in clinical practice. Tumours expressing progesterone receptor (PR) and/or estrogen receptor (ER) are considered hormone receptor-positive breast cancers, while in triple-negative breast cancer (TNBC), tumours do not express any of these receptors. Just as in the rest of the world, invasive ductal carcinoma is the commonest histological type of breast cancer in Africa [12, 13]. Acute T-cell leukemia (ALT) exhibits a very invasive pattern of progression associated with a reduced overall survival due to factors such as chemoresistance and immunosuppression [14, 15]. Due to high mortality associated with the aforementioned cancers, there is the need for agents with high efficacy (and few adverse effects) to help in managing these conditions.

During the past 18 years, cancer treatment strategies have markedly shifted from the use of cellularly-targeted therapies to the elaboration of molecularly-targeted drugs. However, using a single molecularly-targeted agent would often not yield good clinical response. Therefore, the use of combination therapy targeting multiple molecular sites could be of benefit in managing cancers [16]. Medicinal plants used to manage cancers traditionally have been shown to possess different properties such as antioxidant, anti-inflammatory, cytotoxic, immunostimulant, anti-bleeding, providing minerals, vitamins, enzymes, and micronutrients to the body, etc. [17, 18]. Therefore, the use of medicinal plant (that are combinations of active phytochemicals, micro and macro elements) with established and promising antioxidant, anti-inflammatory and cytotoxic potentials could represent an alternative to conventional cancer chemotherapy in Africa [19].

Although questioned by some authors [20], it is generally reported in the literature that up to 80% of the population in Africa depend on traditional medicines for their primary healthcare needs [21]. In Ghana, there have been efforts to integrate herbal medicines into the conventional health care system [22]. The interest for herbal products is also on the rise even in developed countries such as the United States of America, where approximately 20% of people use herbal products for various health conditions [23]. However, many phytotherapies remain poorly studied.

*Nymphaea lotus* L. (*N. lotus*), from the family Nymphaeaceae (water lily family), is a perennial aquatic flowering plant. It is native to Egypt and grows in various parts of Central Africa, West Africa, and Madagascar [24, 25]. *N. lotus* is known to possess numerous ethnomedicinal properties and often used in the treatment of rheumatic pains, tumours and cancers [26–30]. Reports of phytochemical analysis showed that the major bioactive metabolites in *Nymphaea* species are flavonoids and phenolic compounds, and that *N. lotus* contains very special macrocyclic flavonoids [31, 32]. Based on earlier studies, one can say that *N. lotus* would be a good source of new isolates with high antioxidant, anti-inflammatory and cytotoxic potentials.

Secondary metabolites found in plants that have been shown to be pharmacologically active include flavonoids, alkaloids, tannins, glycosides and sugars, among others [33]. Interaction between secondary metabolites and micro and macro elements in plants are known to either decrease or enhance bioavailability of these secondary metabolites [34]. Conversely, toxic heavy metals that are environmental pollutant from the soil, water or air can be absorbed by medicinal plants at high concentrations, and this could partly explain reported toxic effects of some plants [35]. The World Health Organization therefore recommends systemic determination of heavy metal content and maximum permissible levels of some heavy metals (cadmium, arsenic, and lead) in raw consumable plant materials [36].

The aim of the current study was to determine the in-vitro antioxidant potential, in-vivo anti-inflammatory activity and the cytotoxic property of the hydro-ethanolic extract of the leaves of *N. lotus* on Jurkat (leukaemia) and MCF7 (breast cancer) cell lines, and Chang liver (normal) cell line. Also, this study sought to determine and relate the total phenol, total flavonoid and the macro/micro-elemental constituents of the leaves of *N. lotus* to its pharmacological activities.

## Methods

### Plant collection and extraction

The plant, *N. lotus,* was collected, following the local legislation, in August 2015 from the wild in Koumassi (Latitude: 5° 16′ 60.00“ N; Longitude: -3° 58’ 59.99” W), a suburb of Abidjan (Cote d΄Ivoire). No permission was required to collect these plants. The plant was identified by Mr. Digbeu Luc Gouda (a herbalist) and authenticated by Dr. Cindy Kitcher at the Department of Pharmacognosy and Herbal Medicine (School of Pharmacy, University of Ghana), where a voucher specimen was placed (voucher number PH/2015/001). Plant extraction was performed as previously described [37], with slight modification. Briefly, the fresh leaves of *N. lotus* were separated from the other parts of the plant, dried at room temperature for 2 weeks and then ground into a powder. The powder was then dried again in an incubator at 45 °C for 36 h. A mass of 157.5 g of the powdered plant was weighed and dissolved in 20:80 $$ \raisebox{1ex}{$\mathrm{v}$}\!\left/ \!\raisebox{-1ex}{$\mathrm{v}$}\right. $$ of water and ethanol. Dissolution was in the ratio 1:10 (1 part of powder to 10 parts of solvent). The mixture was macerated with intermittent stirring for 24 h and filtered. Filtrates were pulled together and concentrated using a rotary evaporator (Rotavapor, Switzerland) at 40 °C. The resulting concentrate was freeze-dried. The powder obtained (referred to as NLE) was kept in an air-tight container and stored at room temperature.

### Phytochemical screening

Tests for the presence of phytochemicals (triterpenoids, saponins, flavonoids, tannins, phenolic compounds and alkaloids) contained in the NLE extract were conducted according to protocols previously reported [38].

### Total flavonoid content assay

The total flavonoid content of NLE extract was determined as previously described [39]. Briefly, a mass of 100 μg of different concentration of NLE extract was placed in a 96 well plate, 2% aluminium chloride was added to each well and incubated at room temperature. Absorbances were then read at 420 nm. Total flavonoid was evaluated as quercetin equivalent (mg/ml).

### Total phenolic assay (modified Folin-Ciocalteau method)

The total phenol content of NLE extract was determined as previously described [39]. Briefly, aliquots (10 μl) of NLE extract and gallic acid of different concentrations were placed in Eppendorf tubes. Distilled water (0.79 ml) and Folin-cicocalteau reagent (~ 50 μl) were subsequently added and mixed totally. The mixture was afterwards incubated at room temperature for 8 min. After incubation, Na_2_CO_3_ solution (150 μl) was added, mixed and again incubated at room temperature for 2 h. Aliquots were put in well-plates, and absorbance read at 750 nm. Triplicates concentrations of NLE and gallic acid prepared were used.

### Macro and micro-elements screening: energy dispersive x-ray fluorescence spectroscopic (ED-XRF) measurement

The screening of macro and micro-elements found in *N. lotus* was determined as previously described [40]. Briefly, the powder of *N. lotus* leaves was kept at 60 °C overnight in an oven before pelletisation and subsequent measurement. Triplicate measured samples (4 g/sample) and 0.9 g of a binder (Fluxana H Elektronic BM-0002-1, Licowax C micro powder PM-Hoechstwax, Germany) were homogenized using the RETSCH Mixer Mill (MM301, Germany) and pressed with a manual hydraulic press (SPECAC, UK) to obtain pellets of 32 mm in diameter and 3 mm thickness. These were used for subsequent XRF measurements. The detection limit for light elements (Si, Al, Mg and Na) was in the range of 25–50 ppm. For heavy metals, 1–5 ppm was the limit of detection. Calibration was factory done using international rock standards. Simultaneous measurement and analysis of the elemental contents of the samples of *N. lotus* leaves were performed using a SPECTRO X-Lab 2000 spectrometer.

### Antioxidant assays

#### 1,1-diphenyl-2-picryl hydrazyl radical (DPPH) assay

The DPPH assay was performed as previously described [39]. Briefly, different concentrations of NLE extract were made by threefold serial dilutions starting from an initial concentration of 20 mg/ml. The test samples (100 μL) were added in triplicates to 100 μL of DPPH in well plates and gently shaken. The plate was covered with a foil and incubated in the dark, at room temperature, for 20 min. Absorbances were then read at 517 nm. The amount of sample required to react with one half of the DPPH was expressed as the relative amount of the positive control, butylated hydroxytoluene (BHT), that reacted. Antioxidant activity of samples was expressed as DPPH radical scavenging by the formula:

$$ \%\mathrm{DPPH}\ \mathrm{radical}\ \mathrm{scavenging}=\frac{\mathrm{Sample}\ \mathrm{absorbance}}{\mathrm{Control}\ \mathrm{absorbance}}\ \mathrm{x}\ 100 $$

#### Lipid peroxidation assay

The lipid peroxidation assay was performed as previously described [41]. Briefly, 100 μL of different concentrations of NLE extract, obtained by twofold dilutions, were added to 1 ml of egg lecithin [3 mg/ml] in phosphate buffer. 10 μL of FeCl_3_ [400 mM] and 10 μL of L-ascorbic acid [200 mM] were then added to induce lipid peroxidation. After incubation for 1 h, at 37 °C, 1 ml of 15% trichloroacetic acid (TCA) and 1 ml of 0.375% tert-butyl alcohol (TBA) in 20% acetic acid were added to stop the reaction. The mixture was boiled for 15 min, allowed to cool and centrifuged at 300 g. Absorbance of the supernatant was measured at 532 nm.

#### Reducing power assay

The reducing power assay was performed as previously described [42]. Briefly, 200 μL of NLE extract were added and mixed to 200 μL of potassium ferricyanide. The mixture was incubated at 50 °C for 20 min, and a volume of 200 μL of trichloroacetic acid (TCA) was then added and centrifuged at 300 g for 10 min. Two hundred (200) μL of the supernatant were pipetted into a tube, and 200 μL of distilled water and 40 μL of ferric chloride were added. The blank was a solution with all reagents except the NLE. The resulting solution was incubated for 30 min and pipetted into well plates, after which absorbance was read at 700 nm. Ascorbic acid and BHT were used as positive controls.

### Anti-inflammatory assays

#### Animals care and safety

This research was reviewed and approved by the Ethical and Protocol Review Committee of the College of Health Sciences, University of Ghana (Protocol Identification Number: CHS-Et/M.6-P1.5/2017–2018) and was conducted in accordance with the internationally accepted principles for laboratory animal use and care as found in the US guidelines (NIH publication #85–23, revised in 1985).

Sixty pathogen-free male Sprague-Dawley rats (Hsd: SD strain), aged 2 to 3 months and weighing 150–200 g, were purchased from the Center for Plant Medicine Research (CPMR), Mampong, Eastern Region, Ghana. Animals were housed in stainless steel cages of 2 cubic feet (61 cm × 31 cm × 31 cm) with softwood shavings as bedding. They were maintained under standard laboratory conditions (temperature ~ 25 °C, relative humidity 60–70%, and 12 h light-dark cycle), fed with standard pellet diet (AGRIMAT, Kumasi, Ghana) and allowed access to water ad libitum. Animals were acclimatised under these conditions for 7 days before the experiment. To prevent contamination, animals’ feeding, and water troughs were washed frequently.

#### Paw edema test

The paw edema test was performed as previously described [43]. Briefly, rats were randomly divided into 6 groups of 5 each. Oedema was induced by sub-plantar injection of 100 μL of 1% freshly prepared solution of carrageenan into the right-hind paw of each rat of all groups. Group 1 served as negative control and was administered distilled water; groups 2, 3 and 4 were treated with NLE at 100, 250 and 500 mg/kg of body weight (bw) respectively, 3 days prior to the induction of inflammation. Groups 5 and 6 were given diclofenac (2 mg/kg bw) orally and hydrocortisone succinate (4 mg/kg bw) intraperitoneally 30 min before carrageenan injection. Paw thickness was measured just before the carrageenan injection (initial) and then at 1, 2, 3, 4, and 24 h after carrageenan injection. Increase in paw thickness was measured as the difference in paw thickness at the initial time and paw thickness at respective hours using a plethysmometer.

#### Skin prick test and erythrocyte sedimentation rate (ESR)

The skin prick test was performed, as previously described [44]. Briefly, rats were randomly divided into 6 groups of 5 each and sensitized (group 2, 3, 4, 5 and 6) or not (group 1) by intraperitoneal and subcutaneously injections of albumin (OVA) emulsified in aluminum hydroxide on days 1 and 7. Group 1 (non-sensitized control) and group 2 (OVA-sensitized control) received distilled water orally. In contrast, groups 3, 4 and 5 were pre-treated with daily oral administration of NLE extracts (100, 250 and 500 mg/kg bw) and group 6 with intraperitoneal administration of hydrocortisone succinate (4 mg/kg bw) over 8 days, from day 1. On day 9, two separate areas on the skin of all rats were shaved using a blade and the prick test was carried out on all rats by subcutaneous injection of histamine. The time for the appearance of the swelling was noted and the wheal diameters were recorded for all rats at 1 h and 2 h after histamine injection. The average skin oedema was calculated for each group and expressed as a percentage of the OVA-sensitized control. Percentage of skin oedema relative to that of OVA-sensitized control for each group was calculated according to the formula:

$$ \%\mathrm{Skin}\ \mathrm{oedema}=\frac{\mathrm{oedema}\ \mathrm{in}\ \mathrm{a}\ \mathrm{particular}\ \mathrm{treated}\ \mathrm{group}}{\mathrm{oedema}\ \mathrm{in}\ \mathrm{sensitized}\ \mathrm{control}\ \mathrm{group}}\ \mathrm{x}\ 100 $$

Rats were then sacrificed by cervical dislocation and the whole blood was collected by cardiac puncture into citrate tubes under sterile conditions. The erythrocyte sedimentation rate was determined as previously described [45, 46]. Briefly, the Westergren pipette was gently inserted into the citrate tube, and by capillary action, the anticoagulated blood rose to the 0 mm mark to the top of the pipette. The pipette was placed into a stand and fixed in a vertical position for 1 h. The sedimentation rate was then measured in mm/h by reading from the calibrations on the pipette.

### In-vitro cytotoxic activity of NLE

#### Cell culture and treatment

Cell culture and treatment were performed as previously described [47]. Jurkat, MCF-7 and Chang liver cell lines, provided by the Noguchi Memorial Institute for Medical Research, University of Ghana, were cultured in EMEM containing 10% FBS and 1% penicillin/streptomycin and all cells were maintained at 37 °C, 100% relative humidity, 5% CO_2_, 95% air, and culture media changed twice a week. The cells were subcultured when they reached 80% confluence. For the treatment of adherent cells (MCF-7and Chang Liver cells), well plates at a density of 1 × 10^4^ cells/well were seeded with 100 μl of cell suspension and incubated for 24 h. The extract concentrations ranged from 62.5 to 1000 μg/ml and curcumin (positive control) from concentrations of 0 to 36.84 μM were then added to the cells and incubated for 48 h. For the treatment of suspension cells (Jurkat), 1 × 10^4^cells/well were seeded and varying concentrations of the extract (62.5 to 1000 μg/ml) and curcumin (0 to 36.84 μM) were added in the wells. The plates were incubated at 37 °C in a humidified atmosphere containing 5% CO_2_.

#### MTT assay

Cytotoxic effects of NLE and curcumin on cell lines were determined using an MTT assay as previously described [47]. Briefly, for adherent cells, after 48 h of treatment incubation, 15 μl of MTT [5 mg/ml] in phosphate-buffered saline (PBS) were added and incubated at 37 °C for 4 h. MTT was flicked off and the formed formazan crystals solubilised in 100 μl of DMSO. Absorbance was measured at 570 nm, using a microplate spectrophotometer (Tecan Infinite M200 Pro plate reader, Austria). For suspension cells (Jurkat), after 48-h treatment, the medium was discarded and 20 μl/well of MTT solution [5 mg/ml] added. The plate was incubated for 3 h at 37 °C. Finally, 20 μl of isopropanol was added to each well and the plate read using a spectrophotometer at a wavelength of 590 nm. The percentage of cell inhibition was determined using the formula:

$$ \%\mathrm{Cell}\ \mathrm{growth}\ \mathrm{inhibition}=100-\left(\frac{\mathrm{Mean}\ \mathrm{absorbance}\ \mathrm{of}\ \mathrm{treated}\ \mathrm{cells}}{\ \mathrm{Mean}\ \mathrm{absorbance}\ \mathrm{of}\ \mathrm{untreated}\ \mathrm{cells}}\ \mathrm{x}\ 100\right) $$

The experiment was done in triplicate. A graph of mean percentage viability against concentration was plotted, using Graph pad prism 5 and the IC_50_s of curcumin and the extract (where applicable) were determined.

### Statistical analysis

Percentage inhibition against the log of concentration was plotted for each sample tested, and 50% inhibitory concentration (IC_50_) interpolated from the curves. IC_50_ values were determined using Graph Pad Prism software version 5.0 for Windows (GraphPad Software, San Diego, CA, USA) and expressed as IC_50_ mean value (± SEM). Graphs were plotted using Sigma Plot for Windows Version 11.0 (Systat Software Inc., Germany). A duplication method was used before the actual analysis of the micro and macro-elements. The method employs three-axial geometry, thereby reducing background noise by radiation polarisation.

## Results

### Phytochemical, total flavonoid and total phenolic contents of NLE

The phytochemical screening of NLE revealed the presence of triterpenoids, saponins, flavonoids, tannins and phenolic compounds and the absence of alkaloids. The total flavonoid content of NLE was expressed as quercetin equivalent and extrapolated from the standard curve of quercetin (Fig. 1.1-A). Concentrations of 0.31 and 0.63 mg/ml of NLE contained 1.8 and 2.2 g of total flavonoid per 100 g of quercetin equivalent, respectively (Fig. 1.1-B). In the other hand, the total phenolic content of NLE, expressed as gallic acid equivalent, was extrapolated from the standard curve of gallic acid (Fig. 1.2-A). At concentrations of 2.5; 5.0 and 10.0 mg/ml of NLE, the total phenolic contents determined were 10.5; 11.8 and 10.6 g /100 g of gallic acid equivalent, respectively (Fig. 1.2-B).

**Fig. 1:**
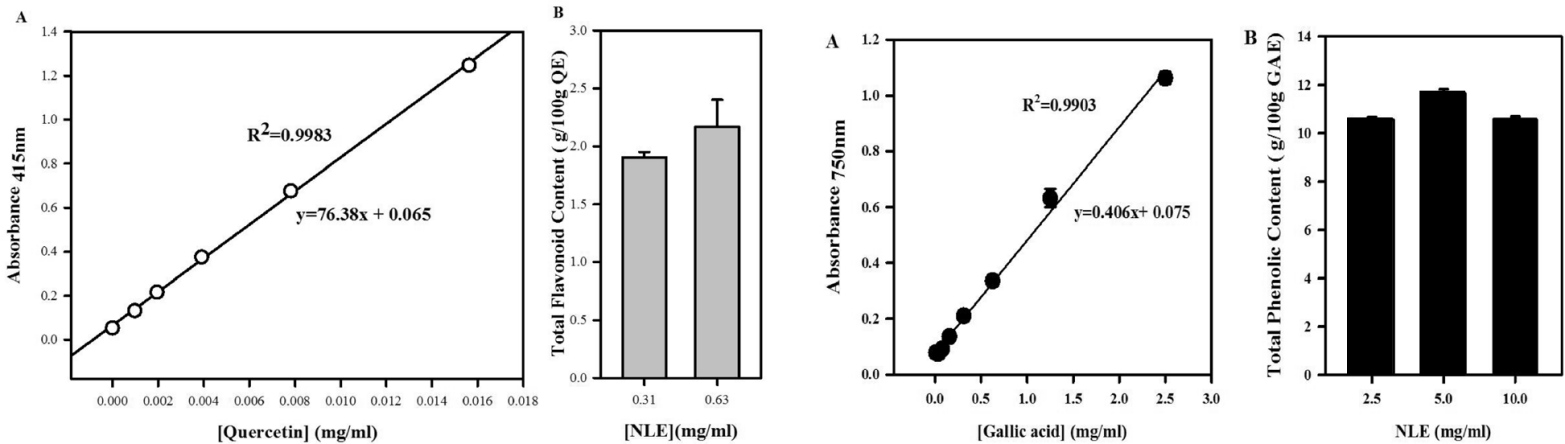
Total flavonoids and total phenolic contents of the hydro-ethanolic extract of *Nymphaea lotus* (NLE) leaves. Panel 1.1: Total flavonoid content of NLE (2.5 mg/ml – 10 mg/ml) expressed as quercetin equivalent (QE). Panel 1.2: Total phenolic content of various concentrations of NLE (2.5 mg/ml- 10 mg/ml) expressed as gallic acid equivalent (GAE). Each point represents the mean value ± standard error on the mean (SEM), (*n* = 3)

### Micro and macro-elements content of NLE

A total of nine 9 macro-elements and 11 micro-elements were identified and quantified in NLE samples (Table 1). The macro-elements of mean values between 414 and 38,403 ppm identified were magnesium (Mg), aluminum (Al), silicon (Si), phosphorus (P), sulphur (S), potassium (K), calcium (Ca), manganese (Mn), and iron (Fe). While the micro-elements of mean values between 2 and 40 ppm were copper (Cu), zinc (Zn), rubidium (Rb), strontium (Sr), yttrium (Y), zirconium (Zr), niobium (Nb), silver (Ag), cadmium (Cd) and thorium (Th). The content of the elements quantified in *N. lotus* leaves decreases in the order: Si > K > Ca > Mg > Al > Fe > S > P > Mn > Ag > Zn > Rb > Sr > Cd > Th > Zr > Cu > Nb > Y.

**Table 1:** Content and limit of detection (ppm) of micro and macro-elements in *Nymphaea lotus* leaves

Elements	Mean ± 3σ (ppm)	Limit of detection LOD (ppm)
Mg	11,433 ± 0.43	1100
Al	7733 ± 0.01	95
Si	38,403 ± 0.036	160
P	2959 ± 97	10
S	3225 ± 68	13
K	21,500 ± 0.021	16
Ca	13,800 ± 0.009	17
Mn	414 ± 28	5
Fe	5859 ± 87	12
Cu	6 ± 3	2
Zn	33 ± 3	1
Rb	28 ± 1	1
Sr	19 ± 1	1
Y	2 ± 1	1
Zr	9 ± 2	1
Nb	3 ± 2	1
Ag	40 ± 8	2
Cd	16 ± 9	2
Pb	–	2
Th	15 ± 5	2

Macro and micro-elements content were measured on pellets of *N. lotus* leave samples (32 mm diameter × 3 mm thickness) using energy dispersive x-ray fluorescence (ED-XRF) spectroscopy (SPECTRO X-Lab 2000 spectrometer). Measurements were done in triplicate and data presented are expressed in ppm and are mean value ±3σ. The limit of detection for light elements (Si, Al, Mg, and Na) was in the range of 25–50 ppm and 1–5 ppm for heavy metals

### Antioxidant activities of NLE

**Fig. 2:**
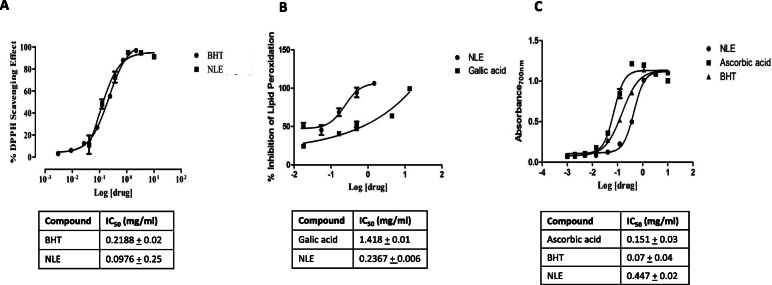
Antioxidant activities of the hydro-ethanolic extract of *Nymphaea lotus* (NLE). **a**: 1,1-diphenyl-2-picryl hydrazyl radical (DPPH) radical scavenging activity, with each point representing mean value ± standard error on the mean (SEM), (n = 3). **b**: Inhibition of lipid peroxidation by NLE and gallic acid, with each point representing mean ± SEM (n = 3). **c**: Reducing power of NLE, Ascorbic acid and butylated hydroxytoluene (BHT), with each point representing mean ± SEM (n = 3)

The extract and BHT exhibited a concentration-dependent scavenging activity of DPPH (Fig. 2a). The concentration of NLE required to inhibit 50% of free radicals (IC_50_) was 0.0976 ± 0.25 mg/ml compared to 0.2188 ± 0.02 mg/ml obtained for BHT (positive control). The difference in IC_50_ values between the NLE and BHT was statistically significant (*p* < 0.001) and NLE was found to be about two-fold more potent than BHT, in scavenging DPPH radicals. Also, both NLE and gallic acid caused a concentration-dependent inhibition of lipid peroxidation (Fig. 2b). The IC_50_ values determined for NLE and gallic acid were 0.2367 ± 0.006 and 1.418 ± 0.01 mg/ml, respectively. The difference in IC_50_ values between the NLE and gallic acid was statistically significant (*p* < 0.001) and NLE was about six-fold more potent in inhibiting lipid peroxidation than gallic acid. The extract, ascorbic acid and BHT showed a concentration-dependent reducing power (Fig. 2c). IC_50_ determined were 0.447 ± 0.02; 0.151 ± 0.03 and 0.07 ± 0.04 mg/ml, for NLE, ascorbic acid and BHT, respectively. There were significant differences (p < 0.001) between the IC_50_ values of NLE, ascorbic acid and BHT. However, NLE was about 3 and 6-fold less potent than ascorbic acid and BHT, respectively, in reducing Fe^3+^ to Fe^2+^.

### Anti-inflammatory activities of NLE

#### Rat paw oedema reduction

**Fig. 3:**
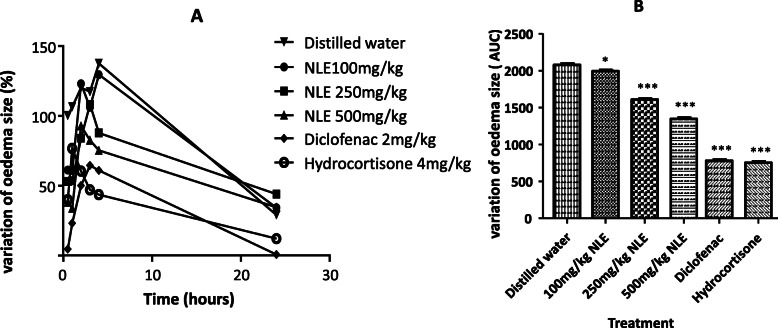
Anti-inflammatory activities of the hydro-ethanolic extract of *Nymphaea lotus* leaves (NLE, 100, 250 and 500 mg/kg, p.o.), diclofenac (2 mg/kg) and hydrocortisone (4 mg/kg) on the paw oedema size in normal SD rats. Chart **a** shows the time course effects over a 24-h period; chart **b** shows the total percentage paw oedema’s area under the curve (AUC) over the 24-h period. Data are mean value ± standard error on the mean (SEM), (*n* = 5). *p < 0.05, ***p < 0.001 when compared to the negative control group, using one-way ANOVA followed by a Dunnett’s Multiple Comparison Test

Sub-plantar injection of carrageenan in the paw induced oedema (in all rats) which size increased with time till 4 h and then spontaneously decreased till 24 h after injection (Fig. 3). However, rats pre-treated with NLE or standard drugs (diclofenac and hydrocortisone) exhibited significantly reduced paw oedema sizes at all points measured in time when compared to the negative controls. These effects of NLE and standard drugs on the oedema size were time- and dose-dependent and were statistically significant among groups (*p* < 0.05 and *p* < 0.0001 respectively).

#### Skin prick test

**Fig. 4:**
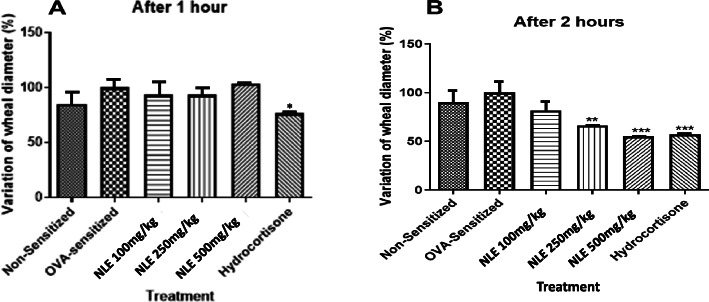
Anti-inflammatory activities of the hydro-ethanolic extract of *Nymphaea lotus* leaves (NLE, 100, 250 and 500 mg/kg, p.o.) and hydrocortisone (4 mg/kg) on wheal diameter in ovalbumin (OVA)-sensitized SD rats, 1 and 2 h after intradermal injection of histamine. Data are mean value ± standard error on the mean (SEM), (n = 5). **p < 0.01, ***p < 0.001 when compared to the negative control group, using one-way ANOVA followed by a Dunnett’s Multiple Comparison Test

Intradermal injection of histamine induced the formation of a wheal on the skin of all rats (Fig. 4). An hour after histamine injection, only rats pre-treated with hydrocortisone presented a significantly reduced wheal diameter, when compared to the negative control group (*p* < 0.05). However, 2 h after histamine injection, rats pre-treated with NLE or the standard drug (hydrocortisone) exhibited a significantly reduced wheal diameter, as compared to the negative control. These effects of NLE and the standard drug on the wheal diameter were time- and dose-dependent and were statistically significant at NLE 250 and 500 mg/kg (p < 0.05 and p < 0.0001).

#### Erythrocyte sedimentation rate (ESR)

**Fig. 5:**
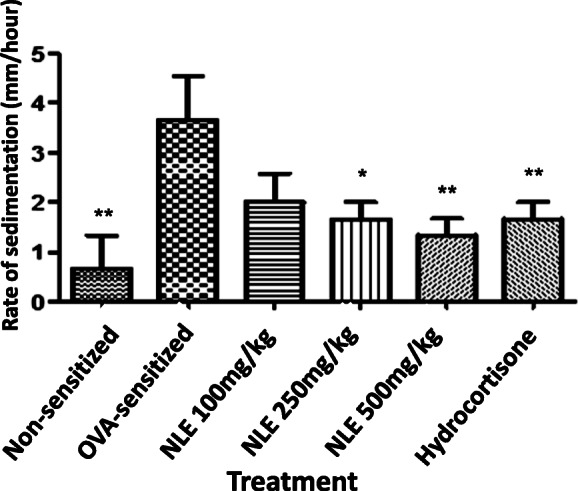
Anti-inflammatory activities of the hydro-ethanolic extract of *Nymphaea lotus* leaves (NLE, 100, 250 and 500 mg/kg, p.o.) and hydrocortisone (4 mg/kg) on the rate of sedimentation of erythrocyte (ESR) from blood samples of ovalbumin (OVA)-sensitized SD rats. Data are mean value ± standard error on the mean (SEM), (n = 5). *p < 0.05, **p < 0.01 when compared to the negative control group, using one-way ANOVA followed by a Dunnett’s Multiple Comparison Test

Control rats sensitised by ovalbumin showed a significantly increased ESR (*p* < 0.01) when compared to the non-sensitized rats. Ovalbumin-sensitized rats treated with increasing doses of NLE exhibited a dose-dependent decrease of the ESR. However, this NLE-induced decrease of ESR was significant at 250 and 500 mg/kg (p < 0.05 and p < 0.01 respectively). Also, ESR was significantly decreased (p < 0.01) in rats treated with hydrocortisone when compared the control sensitised rats (see Fig. 5).

### Effect of NLE on MCF-7, Jurkat and Chang liver cell lines proliferation

**Fig. 6:**
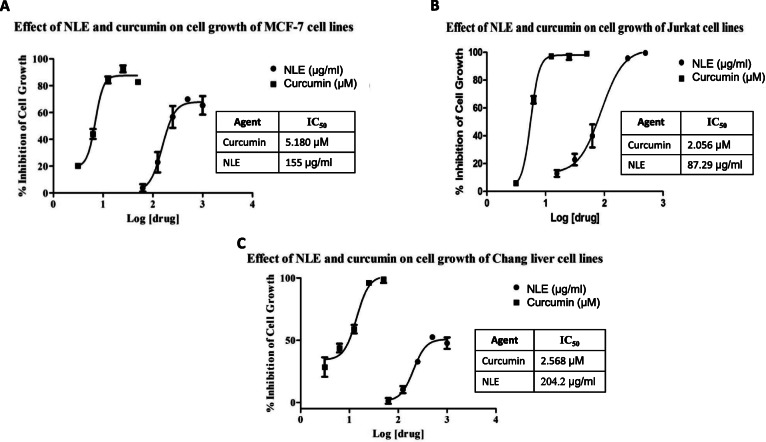
Cytotoxic activities of the hydro-ethanolic extract of *Nymphaea lotus* leaves (NLE) and curcumin on cell growth of breast cancer (MCF-7) cell line (**a**), leukaemia (Jurkat) cell line (**b**) and Chang liver cells (**c**). Each point represents the mean value ± standard error on the mean (SEM), (n = 3)

The effect of NLE on MCF-7, Jurkat and Chang liver cell lines proliferation is presented in Fig. 6. The extract and curcumin (positive control) exhibited concentration-dependent inhibition of MCF-7 with IC_50_ values of 5.180 and 155 μg/ml for curcumin and NLE, respectively. The difference between the IC_50_ values of NLE and curcumin were statistically significant (*p* < 0.001). Both NLE and curcumin inhibited the growth of Jurkat cells in a concentration-dependent fashion with IC_50_ values of 2.056 and 87.29 μg/ml for curcumin and NLE respectively and the difference was found to be statistically significant (p < 0.001). The extract and curcumin exhibited a concentration-dependent inhibition on Chang liver cell growth with IC_50_ values of 204.2 and 2.568 μg/ml for NLE and curcumin respectively and this difference was statistically significant (p < 0.001).

## Discussion

There is a growing body of evidence that suggests that oxidative stress and inflammation are two primary mechanisms leading to the initiation and development of cancers. This study sought to contribute to the management of cancer by investigating the antioxidant, anti-inflammatory and cytotoxic activities of *N. lotus* (a medicinal plant traditionally used to manage cancer patients) in relation to its phytochemical and elemental constituents.

The phytochemical screening of the hydro-ethanol extract of *N. lotus* leaves (NLE) revealed the presence of triterpenoids, saponins, flavonoids, tannins and phenolic compounds and the absence of alkaloids. Also, the determination of the total phenol and total flavonoids contents of NLE extract revealed appreciable amounts of these two secondary metabolites. The presence of saponin, triterpenoids and considerable amounts of flavonoids and phenolic compounds in our extract could explain the antioxidant, anti-inflammatory and cytotoxic activities of NLE, as similarly reported by previous studies on aqueous and acetone extracts of *N. lotus* [48–50]. Likewise, these findings are consistent with a study showing the presence of vitamins A, C and E in *N. lotus* leaves [51]. Previous reports showed the presence of alkaloids in the chloroform, ethyl acetate, n-butanol, acetone and aqueous extracts of *N. lotus* leaves, in addition to the presence of phenolic compounds (flavonoids, coumarins and tannins), and sterols [50, 52]. The absence of alkaloids in our extract (NLE) could be due to solvent system used for the extraction or the geographical location of plants collected. Our choice for the hydro-ethanolic system was because we wanted to mimic, as much as possible, the traditional preparation of *N. lotus.*

The elemental content of NLE sample was measured by energy dispersive x-ray fluorescence spectroscopy (ED-XRF). It is a fast, reliable and powerful analytical tool for the determination of the elemental composition of diverse materials (particularly for high energy elements) and does not produce waste and requires no chemical reagents [53]. A total of nine 9 macro and 11 micro-elements were identified and quantified in the NLE sample (Table 1). The content of various elements analyzed in *N. lotus* leaves decreases in the order: Si > K > Ca > Mg > Al > Fe > S > P > Mn > Ag > Zn > Rb > Sr > Cd > Th > Zr > Cu > Nb > Y. Macro-elements such as magnesium (Mg), phosphorus (P), sulphur (S) and micro-elements such as manganese (Mn), copper (Cu) and zinc (Zn) present in NLE sample have been reported to be relevant elements as anti-cancer adjuvants [54, 55]. These elements are known to play important roles in cellular metabolism, as co-factors in numerous enzymatic activities, or as important components of structural proteins such as hormones involved in cancer therapy [56]. Copper and zinc have been reported to be involved in the elimination of free radicals through cascading enzyme systems [57]. Thus, the presence of these elements in NLE could support the traditional use of *N. lotus* as an anticancer medicinal plant and also explain the antioxidant, anti-inflammatory and cytotoxic activities of NLE reported in this study. Moreover, cadmium (Cd), a toxic heavy metal, was detected in the NLE sample. Lead was below the detectable limit. These findings are almost similar to results reported [51] using a flame photometer and an atomic absorption spectrophotometer (AAS). This finding proved that results obtained using ED-XRF or AAS methods are not significantly different [58]. However, it is important to note that acute or chronic exposure of an organism to these toxic elements (which are usually contaminants from the environment) present in medicinal plants could result in the development of unwanted toxicopathies [59]. Therefore, the presence of these toxic elements (but not *N. lotus* itself) could explain the toxic effects of the hydro-ethanolic and hydro-methanolic extracts of *N. lotus* reported in the literature. These toxic effects were characterised by an induction of a chromosomal aberration in rat lymphocytes, an increase of aberrant sperm cells and somatic genotoxicity [49, 60].

The antioxidant activity of NLE extract was determined using DPPH radical scavenging assay, lipid peroxidation inhibition assay and reducing power assay. The DPPH radical scavenging assay revealed that NLE was about two times more potent than BHT in scavenging DPPH. This result shows that NLE has a strong antioxidant potential, and that could be attributed to its phenolic, flavonoid and elemental contents. A correlation between phenolic compounds, micro-element content and antioxidant activity has been reported in the literature [61–63]. In the current study, NLE was also found to be about six-fold more potent in inhibiting lipid peroxidation than gallic acid. The extract’s ability to inhibit lipid peroxidation could be attributed to the presence of lipid-soluble antioxidant compounds, hence, could donate protons to stabilize lipid radicals or exquisitely scavenges lipid peroxyl radicals, thereby, terminating subsequent chain propagation reactions [64]. In a reducing power assay, the oxidative form of iron (Fe^3+^) in ferric chloride is converted to ferrous (Fe^2+^) by antioxidant compounds. The extract was not as potent as ascorbic acid or BHT in reducing Fe^3+^ to Fe^2+^. In a similar study, the hydro-alcohol and chloroform extracts of *Nyctantes arbour* were found to be as potent as ascorbic acid in reducing power property [65].

The anti-inflammatory activity of NLE extract was measured using carrageenan-induced rat paw oedema assay, skin prick test and erythrocyte sedimentation rate assay. Carrageenan-induced rat paw oedema model is an appropriate test commonly used in screening of anti-inflammatory agents. The biphasic acute inflammation induced by carrageenan is characterized by the release of pro-inflammatory mediators such as kinins, histamine and serotonin, in the first phase [66], followed by the release of prostaglandins-like substances, the main mediators causing the acute inflammation [67]. This later phase is responsive to clinically useful anti-inflammatory agents [68]. The extract significantly decreased paw oedema sizes in a dose-dependent manner, thus, might be containing some anti-inflammatory compounds causing the inhibition of prostaglandin-induced inflammatory pathway. Subcutaneous injection of histamine (or other allergens) causes mast cells to degranulate and release histamine and other mediators, following the cross-linkage of specific IgE bound to their surface receptors. This leads to a triple response characterized by a capillary dilatation (causing a red spot), an arteriolar dilatation mediated by axon reflex leading to a flare (causing the redness in the surrounding area) and an exudation of fluid from venules and capillaries (causing the formation of a wheal), which wheal can be quantitated by measuring its diameter [44]. Thus, the skin prick test was used to measure the anti-inflammatory effect of NLE on the immunoglobulin E (IgE) response to ovalbumin (OVA) and on the vasodilatory activity of a preformed mediator, histamine, in sensitized rats. The extract significantly decreased wheal diameters in a dose-dependent manner, thus, might be containing anti-inflammatory compounds that could also block mast cells degranulation and the release histamine (or other mediators). Erythrocyte sedimentation rate assay measures the distance that erythrocytes have sedimented (under the influence of gravity) after 1 h in a vertical column of anticoagulated blood. The main factor influencing and directly correlating with the sedimentation rate is the amount of fibrinogen in the blood. The extract significantly decreased the ESR in a dose-dependent manner, thus, might be containing anti-inflammatory compounds that could block the synthesis of fibrinogen. Altogether, these results showed a consistent anti-inflammatory activity of NLE extract. These findings are consistent with the traditional use of *N. lotus* as anti-inflammatory medicine [69] to treat arthritis and rheumatic pains [70].

Invasive ductal carcinoma is the commonest histological type of breast cancer in Africa and acute T-cell leukemia exhibits a very aggressive course of progression with poor prognosis associated with shorter overall survival. Therefore, there is a need to discover new effective agents against these two types of cancer. MCF-7 is a human adenocarcinoma breast cancer cell line with estrogen, progesterone and glucocorticoid receptors, while Jurkat cell line was derived from human acute T-cell leukaemia. These two cell lines are excellent models for screening anticancer (cytotoxic) agents. The cytotoxic activity of NLE extract was determined using an MTT assay. Both NLE and curcumin (positive control) exhibited cytotoxic activities on MCF-7 and Jurkat cell lines, however, NLE was not as potent as curcumin. This difference could be explained by the fact that curcumin is a pure isolated compound and NLE is a crude extract containing several active compounds which could have antagonistic effects on each other. The cytotoxic activity of NLE revealed some cell-specificity for Jurkat cell line, as shown by the IC_50_ values of NLE for Jurkat and MCF-7. Interestingly, NLE showed less cytotoxic activity on Chang liver cells (normal cell line) when compared to the cancer cell lines (Jurkat and MCF-7). In the opposite, curcumin rather showed a potent cytotoxic activity on Chang liver cell line. These results corroborate similar antiproliferative studies on the cytotoxic activities of *Nymphea lotus* (whole plant), *Nymphea alba* and *Nymphaea mexicana* [27, 71] and could be attributed to the phytochemical and elemental contents of *N. lotus* leaves and therefore, could justify its traditional use as cancer treatment. Further bioactivity-guided fractionation studies would be necessary to isolate the active compounds responsible for the aforementioned activities.

## Conclusion

Our results showed that the hydro-ethanolic extract of *N. lotus* leaves possesses high antioxidant potential, anti-inflammatory property and cytotoxic activity on MCF-7 and Jurkat cancer cell lines. These properties could be attributed to the presence of appreciable amounts of total phenol and flavonoids, as well as to the presence of macro/micro-elements such as magnesium, phosphorus, sulphur, manganese, copper and zinc. However, the presence of heavy metals such as cadmium detected in *N. lotus* leaves could explain the toxic effects of the hydro-ethanolic extract of *N. lotus* previously reported in the literature. Altogether, our findings could explain the use of *N. lotus* leaves in the traditional management of cancer patients and justify the use of this plant as a source for the discovery of new anticancer agents with cytotoxic, antioxidant and anti-inflammatory properties.

## Data Availability

The data used to support the findings of this study are available from the corresponding author upon request.

## Abbreviations

ANOVA: Analysis of variance
AUC: Area under the curve
BHT: Butylated hydroxytoluene
DPPH: 2, 2 diphenyl-1-picrylhydrazyl
ED-XRF: Energy dispersive x-ray
ESR: Erythrocyte sedimentation rate
IC_50_: Inhibitory concentration 50
MTT: 3-(4,5-dimethylthiazol-2-Yl)-2,5-diphenyltetrazolium bromide
NCDs: Non-communicable diseases
NLE: *Nymphaea lotus* ethanolic extract
OS: Oxidative stress
SEM: Standard error on the mean
SD: Sprague-Dawley (rats)
WHO: World health organisation
